# Skeletochronology and Paleohistology of *Hyposaurus rogersii* (Crocodyliformes, Dyrosauridae) from the Early Paleogene of New Jersey, USA

**DOI:** 10.3390/ani11113067

**Published:** 2021-10-27

**Authors:** Rodrigo A. Pellegrini, Wayne R. Callahan, Alexander K. Hastings, David C. Parris, John D. McCauley

**Affiliations:** 1 New Jersey State Museum, P.O. Box 530, Trenton, NJ 08625-0530, USA; callahans2@msn.com (W.R.C.); David.Parris@sos.nj.gov (D.C.P.); njtntm@gmail.com (J.D.M.); 2 Science Museum of Minnesota, 120 W. Kellogg Blvd., Saint Paul, MN 55102, USA; ahastings@smm.org

**Keywords:** skeletochronology, paleohistology, Dyrosauridae, *Hyposaurus*, external fundamental system, sexual size dimorphism, bone microstructure, swimming ecology

## Abstract

**Simple Summary:**

The way of life and biology of extinct animals can be inferred from growth marks and microstructures in their fossil bones. *Hyposaurus* is a genus representative of dyrosaurids, a group of crocodile-like animals that survived the extinction event that killed the dinosaurs but nonetheless has long become extinct. The current study focuses on bone sections from a single individual, extracted from the optimal location in the best bones for such studies. It complements and corrects previous works based on less optimal material, and concludes that the genus was a marine ambush predator rather than a fast-swimming pursuit predator, that the males of the genus grew larger than the females, and that the individual studied was a fully mature 17–18-year-old female.

**Abstract:**

The paleohistology of dyrosaurids is known from a small sample, despite being common fossils and representing a rare lineage of crocodylomorphs that survived the Cretaceous–Paleogene extinction. Their lifestyle has been inferred only from sections of the snout, vertebrae, partial femur, and tibia. To improve this, we conducted a skeletochronological and paleohistological study of midshaft cross-sections of both femora and humeri of a nearly complete *Hyposaurus rogersii* skeleton. We found lamellar-zonal bone that underwent remodeling, evidenced by resorption cavities and abundant secondary osteons within the primary periosteal cortex. The osteons, mostly longitudinally oriented and arranged in circular rows, often anastomose radially along a linear path, resembling radial rows. The medullary cavity is completely open, lacking trabeculae: endosteal deposition is limited to thin lamellae surrounding the cavity. Analysis of cyclical growth marks and the presence of an external fundamental system indicate the specimen was a fully mature adult 17–18 years of age. Comparison of the skeleton to others suggests sexual dimorphism and that it was female. The open medullary cavity, and no evidence for pachyosteosclerosis, osteosclerosis, osteoporosis, or pachyostosis indicate *H. rogersii* was not a deep diver or a fast swimmer in the open ocean but a near-shore marine ambush predator.

## 1. Introduction

The longirostrine crocodylomorph *Hyposaurus* is the only dyrosaurid genus known from North America, except perhaps for dyrosaurid remains not identified to the generic level. These include most notably a possible dyrosaurid partial skull, with an additional mandible and partial braincase from the Maastrichtian Escondido Formation of Mexico [1], and a single distal caudal vertebral centrum from the late Eocene Yazoo Clay of Alabama [2]. Partial remains of *Hyposaurus* have been recovered from the Danian Hornerstown Formation of New Jersey, and the early Danian basal Clayton Formation of Alabama, in addition to a questionable vertebral centrum from spoil piles of the late Paleocene Williamsburg Formation of South Carolina [3]. Most of the *Hyposaurus rogersii* material previously collected consisted of isolated bones, although it also included a few associated remains of partial skulls, vertebrae, limb elements, and osteoderms. In the autumn of 2011, a team from the New Jersey State Museum (NJSM) collected the most complete skeleton of *H. rogersii* recovered to date. Designated NJSM 23368, it was excavated from the basal Hornerstown Formation at the classic Inversand locality in Gloucester County, New Jersey, USA.

Although dyrosaurids in general are otherwise common in the fossil record, and are among the few lineages of crocodylomorphs that survived the Cretaceous–Paleogene extinction, their bone histology is only known from a small sample [4,5]. The first report was published by Buffetaut et al. in 1982 and described the histology of sections of the snout and vertebrae of *Dyrosaurus phosphaticus* [4]. It was not until 2014 that, in the second publication on dyrosaurid histology, Andrade and Sayão described sections of a partial femur and tibia of *Guarinisuchus munizi* [5]. Herein, we present an intraskeletal histoanalysis of the femora and humeri of *H. rogersii* NJSM 23368 to bolster the current knowledge of dyrosaurid life history.

## 2. Materials and Methods

### 2.1. Geological Setting

The Hungerford and Terry Inc. (Inversand) quarry was the last remnant of the greensand marl mining industry that thrived in southern New Jersey beginning in the late 1700s. Located outside the town of Sewell in Mantua Township, Gloucester County, the quarry lies within New Jersey’s inner coastal plain, which preserves marine sediments deposited during the latest Cretaceous and early to mid-Paleogene time. NJSM 23368 was recovered from the basal Hornerstown Formations’ Main Fossiliferous Layer—often called the MFL (see [6] for a detailed stratigraphy of the site). The MFL lies just above a major disconformity separating the Hornerstown Formation from the sub-adjacent late Maastrichtian New Egypt Formation. The age of the basal Hornerstown has been a subject of controversy for many years due to the admixture of both the latest Cretaceous and early Paleogene (Danian) vertebrate and invertebrate fossils. Recent studies (e.g., [7,8]) favor an early Danian age and suggest that it represents, in part, a transgressive lag with reworked Maastrichtian fossils mixed with well-preserved Danian remains.

### 2.2. Specimen

NJSM 23368 is a nearly complete skeleton of *Hyposaurus rogersii*. The bones were in close association, although not articulated. The skull is nearly complete and includes the right premaxilla with teeth, nearly complete right maxilla with teeth, posteroventral left maxilla, nasals, frontals, left lacrimal, partial right lacrimal, right prefrontal, postorbitals, squamosals, parietals, supraoccipitals, exoccipitals, basioccipitals, basisphenoid, jugals, quadrates, quadratojugals, pterygoids, ectopterygoids, and unrestored fragments of the palatines. The overall length of the skull is 49.53 cm and the preorbital length is 29.53 cm. The lower jaw is complete and includes the right and left dentaries, splenials, angulars, surangulars, and articulars. Recovered elements of the axial skeleton include the proatlas, atlas intercentrum, atlas neural arches, left atlas rib, axis with odontoid process, 7 additional cervical vertebrae, and 13 cervical ribs. Further, 12 dorsal vertebrae, first sacral vertebra with attached rib and 13 caudal vertebrae, 26 thoracic ribs, 6 haemal arches, and numerous fragments of gastralia were also recovered. The appendicular skeleton includes the left scapula, coracoids, humeri, radii, ulnae, ischia, ilia, pubes, femora, tibiae, fibulae, radiales, a single ulnare, calcanei, left astragalus, indeterminate pedal elements, 7 metapodials, and 16 phalanges including a single ungual. We also recovered 62 whole or partial osteoderms. Most appear to be dorsal paravertebral osteoderms, and the rest accessory. Four limb bones, paired humeri and femora, were sectioned at the mid-shaft for the present paleohistological study.

The material is only slightly permineralized and is exceptionally well preserved. It was recovered from an unlithified bed of the Hornerstown Formation, a sediment known as “green sand” marl, almost exclusively composed of glauconite. It retains its three dimensions with no appreciable compaction or other deformation, as is typical of specimens from the Inversand Company Mine pit site. Excavation at this site must be exceedingly careful, however, as these fossils can be easily deformed and damaged while wet. Once dry, they are remarkably stable, although still fragile. Preparation in the laboratory is often limited to carefully brushing off any remains of matrix from the surface. Impregnation with a consolidant is seldom a critical necessity but is sometimes done to improve the structural integrity and prevent breaks from expected handling during research. Gluing of breaks is rarely required, and disarticulated elements, such as reptile skulls, are only glued if enough pieces are present for a reconstruction.

### 2.3. Preparation

**Figure 1:**
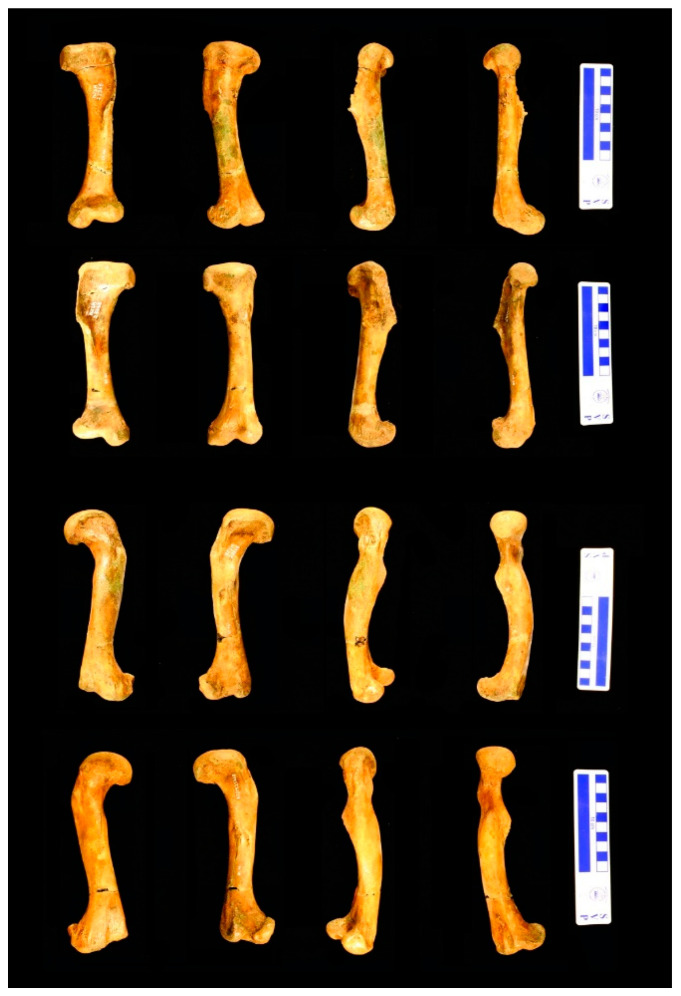
New Jersey State Museum (NJSM) 23368 *Hyposaurus rogersii* elements sectioned. The large scale bar is 10 cm. Left to right: anterior, posterior, lateral, and medial views. Top to bottom rows: left humerus, right humerus, left femur, and right femur.

The femora and humeri were discovered in large pieces with clean breaks, and the diaphyses whole. After the glauconite matrix was brushed and picked off with dental tools, each element was glued together with polyvinyl acetate adhesive. No fillers were applied. Stabilization and impregnation with a thin solution of Paraloid B-72 (an ethyl methacrylate co-polymer, see [9] for suppliers and other details) in ethanol followed, applied by brushing until saturation was detectable. Each limb bone was then measured with calipers, and photographed in detail with a digital camera (Canon EOS Rebel XSi, Canon USA Inc., Lake Success, NY, USA) mounted on a Polaroid MP-4 land camera copy enlargement stand (Figure 1). After photography, 2-part molds of the specimens were made using tin-cure silicone rubber (OOMOO 30, from Smooth-on, Inc., Macungie, PA, USA), and subsequently casts were made from 2-part polyurethane resin (Smooth-Cast 322, Smooth-on Inc., Macungie, PA, USA). Ease Release 200 mold release agent spray (Mann Release Technologies, Macungie, PA, USA) was utilized both in mold and cast making.

**Figure 2:**
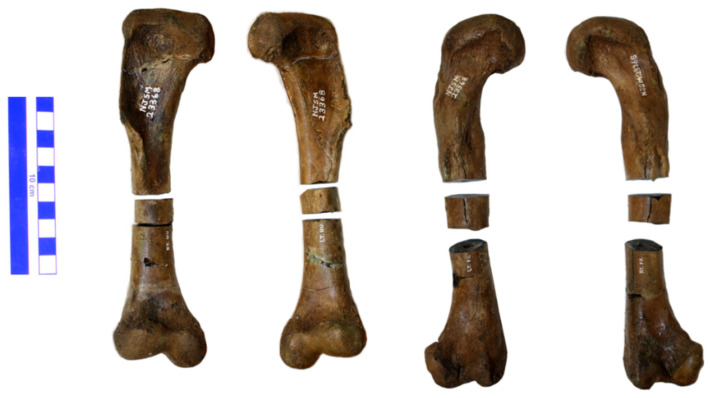
Left to right: NJSM 23368 right humerus, left humerus, left femur, and right femur with extracted samples for thin-sectioning generally in place. The humeri are in anterior view and the femora in posterior view. The large scale bar is 10 cm. This is a composite image.

Whole-diameter samples approximately 1.5 to 2 cm in length were then cut from the mid-diaphysis (Figure 2) using a diamond-bladed wafer rock saw (Graves, Pompano Beach, FL, USA). The resulting epiphyseal specimen fragments were then placed back in the silicone rubber molds, and PB122 PaleoPoxy kneadable 2-part epoxy putty (PaleoBOND, Woodland Park, CO, USA) was used to reconstruct the gap left by the sample extraction. The reconstructed limb elements were then returned to storage with the rest of the NJSM 23368 skeleton.

The samples were tagged and impregnated under vacuum with Epo-tek 301 (Epoxy Technology, inc., Billerica, MA, USA) 2-part epoxy resin, which was colored blue with Kriegrosol Blue Supra Concentrated Powder 05D17-FG (Specialty Coatings & Chemicals, Inc., Los Angeles, CA, USA).

The specimens were then ground roughly flat on lap tables of 20.3-cm-diameter wheels (Hi-tech Diamond, Moorepark, CA, USA and Maxi Lap, Covington Engineering, Redlands, CA, USA) decreasing in grit sizes from 100 to 180. Next, they were ground by hand from 240 to 320, 400, and 600 grit, successively, on Carbimet wet abrasive paper (Buehler, Lake Bluff, IL, USA) until adequately flat for mounting to a glass slide. At this point, they were mounted onto 27 × 46 mm Petrographic Microscope glass slides (Ward’s Natural Science, Rochester, NY, USA) with Loctite 3525 acrylic UV light-curing glue (Henkel Corp., Bridgewater, NJ, USA), under a household black light lamp (15W T8 18” Mol Black Light F15W/BL UV-A 438NM F15T8/BL EIKO T8 Fluorescent obtained from Atlantalightbulbs.com (accessed on 17 February 2011)).

Once the glue cured, the samples were processed on a Hillquist thin-section machine (Hillquist, inc., Denver, CO), which has a diamond-bladed cut-off saw and grinder with a thickness gauge. From this point, the sections were ground thinner and polished by hand on Carbimet wet abrasive paper (Buehler, Lake Bluff, IL, USA), gradually from 240 to 600 grit (as above). Section thickness was evaluated under an American Optical One-Fifty transmitted light compound microscope (American Optical, Buffalo, NY, USA) through the process, under 4 and 10× magnification lenses (Reichert, Buffalo, NY, USA). Once growth marks and other histological structures were clearly visible, the sections were polished with Cerium Oxide #1 powder (Covington Engineering, Redlands, CA, USA) on a 20.3-cm-diameter Maxi Lap cloth polishing lapidary wheel (Covington Engineering, Redlands, CA, USA) that was customized at the factory to rotate at only 380 RPM.

### 2.4. Imaging and Analysis

The thin sections were analyzed under transmitted, polarized, and cross-polarized light, with and without a lambda compensator, with 4×, 10×, and 40× objectives in an Amscope Trinocular Polarizing Microscope with 10×/18 Widefield oculars (obtained through Amscope.com (accessed on 20 August 2010)). To improve contrast and light transmission, occasionally drops of immersion oil (Type A, Cargille Laboratories, Cedar Grove, NJ, USA) were applied to the sections, and micro cover glass installed on top temporarily.

Comprehensive composite digital images of each slide under transmitted and circularly polarized light (CPL) were taken at 5× at the Hard Tissue Research Unit laboratory, Department of Biomaterials and Biomimetics, New York University School of Dentistry, NY, USA. The equipment used was a Leica-Leitz DMRXE Universal Microscope configured with a Marzhauser motorized stage and CPL filters, and PL Fluotar 20/0.50 objective lenses (Leica Microsystems, Buffalo Grove, IL, USA). Image montages were obtained using Syncroscopy Montage Explorer software (Synoptics Inc., Frederick, MD, USA). The devices were calibrated to 1.288 Microns Per Pixel. This setting provided ample resolution to measure structures and growth marks using a 5× lens, thus producing great images for the purpose of our work while making optimum use of our allotted scanning time. Figure 4C–E required higher magnification and were taken with a Canon EOS Rebel T3 camera (Canon USA, Inc., Lake Success, NY, USA) with a Clearshot 600 Digital Camera Adapter System for Canon SLR Digital Cameras from Alexis Scientific (purchased from Alsci.com (accessed on 18 January 2011)), through the Amscope trinocular microscope with 40×, 10×, and 40× objectives, respectively. Figure 1 and Figure 2 were composited in Gimp for illustration purposes from photographs taken using a Canon EOS Rebel XSi camera with a standard Canon EFS zoom lens (18–55 mm).

The measurements of histological structures were taken from the scanned composite images with the Measure tool in Gimp 2.8.16 (GNU Image Manipulation Program, a cross-platform image editor freely available for GNU/Linux, OS X, Windows, and more operating systems from www.gimp.org, accessed on 26 November 2015). The tool produced measurements in pixel number, which were converted to microns by multiplying by 1.288. Lines of arrested growth (LAGs) and cyclical growth marks (CGMs) in general were traced in Gimp at 100% zoom level with a brush size of 20 and hardness of 50, which resulted in 10-pixel-wide lines at their core.

To calculate and compare cortical areas, we first measured the medullary cavity in each element. Diameters were measured at the widest point (diameter a, now the horizontal axis), and at the greatest height at 90° to that line (diameter b, now a vertical axis). Circumferences were calculated from these measurements using Ramanujan’s formula [10]. The cavity areas were obtained using the measure tool in ImageJ (a cross-platform public domain Java image processing program inspired by the US National Institute of Mental Health’s Image program for Macintosh computers). Images of the slides were cropped to show the medullary cavity and its perimeter, then converted to 8-bit grayscale images, and then the threshold was adjusted in over/under mode until the cavity was clearly white, while its perimeter remained unchanged. The scale was set as 1.288 microns per pixel (under the analyze tab), and the wand tool was then used to select the area of the medullary cavity. The measure tool was then used, and results obtained in square microns. When a strut bifurcating the cavity was present, two areas were calculated: one of only the open spaces in the cavity, and another of the entire cavity area (including the strut). To calculate the latter, the original cropped image of the cavity was cleaned up in Gimp by deleting the strut, prior to analysis in ImageJ. The areas of the entire cross-section of each element were similarly obtained, but the image was first cropped to the perimeter of the cortex in Gimp, and then the area inside was painted black. This image was then opened in ImageJ, turned to 8-bit grayscale, scale was set, threshold adjusted, wand tool used, and results quantified in square microns.

## 3. Results

**Figure 3:**
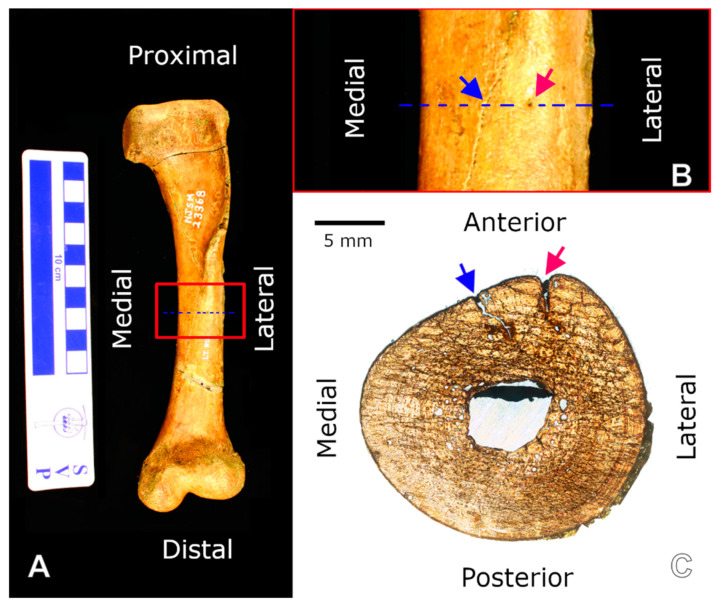
Left humerus of NJSM 23368; (**A**) anterior view. Scale bar is 10 cm; (**B**) A crack (blue arrow) and nutrient foramen (red arrow) are clearly visible in this close-up image of the midshaft (red square in (**A**)). The dotted line indicates the exact location of the thin section pictured in (**C**); (**C**) Slide 2 transverse thin section of the midshaft. Because both the crack and the foramen are located along the top of the section image, it clearly corresponds to the anterior half of the cross-section. The remaining thin sections of all elements in the study were oriented to their respective bone elements similarly through the use of landmarks such as cracks and comparison of the shape of the perimeter of the section to the external morphology of the sampled shaft. Scale bar is 5 mm. A close-up of the nutrient foramen under circularly polarized light (CPL) can be seen in Figure 6E.

The directional terms used to orient the slides relative to their position in each element refer to the position of each element in the skeleton with the element oriented lengthwise parallel to the sagittal and transverse planes. While this would be an unnatural position for the element in a live crocodylomorph, it provides an easy way to address the relative position and orientation from the microanatomy to gross anatomy scales (Figure 3).

The histological descriptions below follow the terminology of Francillon-Vieillot et al. [11].

### 3.1. Paleohistological Description

#### 3.1.1. Left Humerus

Four thin sections were made from the left humerus sample, numbered 1 through 4 sequentially from proximal to distal. They all share essentially the same histology.

**Figure 4:**
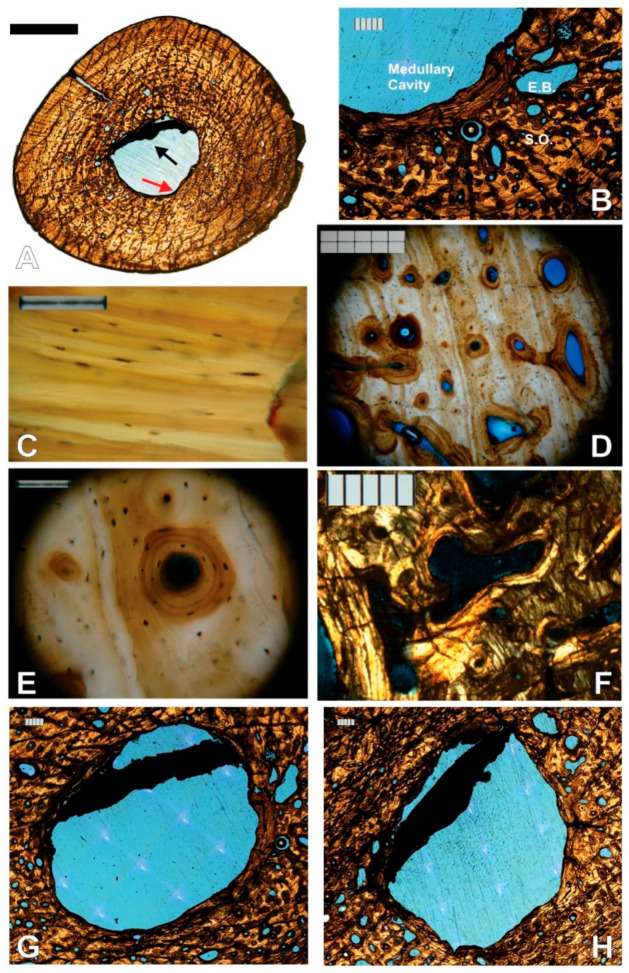
NJSM 23368 left humerus mid-diaphyseal transverse thin-section images. (**A**) Slide 3 of 4, numbered from proximal to distal from the mid diaphysis, under ordinary transmitted light. Top is anterior, right is lateral. Arrows indicate the endosteal, avascular lamellar layer (red) and the structure that divides the medullary cavity (black). Scale bar is 5 mm; (**B**) Close up of endosteal lamellae meeting the deep periosteal cortex in slide 1 under transmitted light. E.B.: Erosion Bay, S.O.: Secondary Osteon. Scale bar is 500 µm; (**C**) Osteocyte lacunae within the endosteal lamellae that surround the medullary cavity under ordinary transmitted light at 40× magnification. Orientation of the long axis is generally along the direction of the bone lamellae. Scale bar is 0.1 mm; (**D**) Longitudinally oriented primary and secondary osteons arranged in circular rows under ordinary transmitted light at 10× magnification. Irregularly shaped osteons and osteons with radial anastomoses are also visible. Osteocyte lacunae are fairly dense. Scale bar is 0.5 mm; (**E**) 40× magnification close up of secondary osteon in the middle of D. The long axis of osteocyte lacunae is also oriented in the direction of the bone lamellae within osteons. Scale bar is 0.1 mm; (**F**) Erosion lacuna developing into irregularly shaped secondary osteon in slide 2 under CPL. Scale bar is 500 µm; (**G**) Medullary cavity in slide 1 under ordinary transmitted light. Scale bar is 500 µm; (**H**) Medullary cavity in slide 2 under ordinary transmitted light. Comparison of the position and shape of the strut structure to A and G. Scale bar is 500 µm.

There is an open medullary cavity that occupies roughly 9% of the cross-sectional area (Supplementary Materials Table S1). It is surrounded by a thin endosteal, avascular lamellar layer (Figure 4A,B). The osteocyte lacunae within the latter are generally oriented in the direction of the bone lamellae (Figure 4C).

As in all elements sampled but the left femur, the medullary cavity is divided by a structure that resembles a strut in the cross-section (Figure 4A,G,H). The strut is fully opaque under transmitted, polarized, cross-polarized (with and without a lambda compensator), and circularly polarized light, which rules out an osseous origin. No histological structures are visible. Throughout the four serial thin sections, advancing distally, it changes position and shape from a straight strut that bisects one-quarter of the medullary cavity, to a curved arc adjacent and almost mirroring the perimeter of the section of the endosteal lamellae to which it connects.

**Figure 5:**
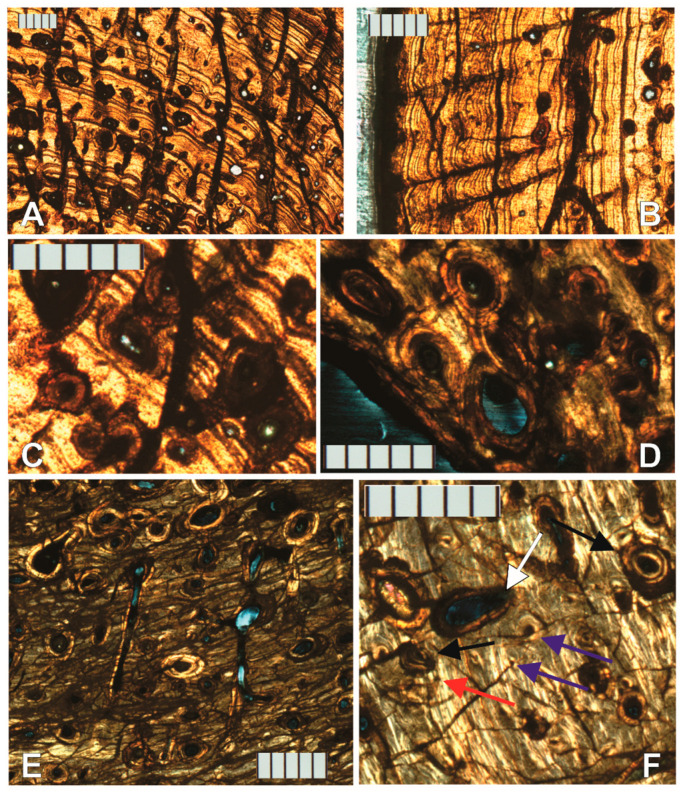
Histological structures in mid-diaphyseal thin sections of the bone of the left humerus of NJSM 23368, *Hyposaurus rogersii*. Images A through C were obtained from slide 3 under ordinary transmitted light. (**A**) Despite cracks in the section, which run from the top to the bottom of the image, growth cycles are readily visible in the lamellar-zonal bone. Longitudinally oriented secondary osteons arranged in circular rows dominate, but primary vascular canals and primary osteons are also common. Radial anastomoses from several of these vascular canals can be seen in this image as well; (**B**) Radial simple primary vascular canals near the periosteal margin; (**C**) First-, second-, and third-generation secondary osteons in the deep cortex; (**D**) First-, second-, and third-generation secondary osteons near the endosteal margin in slide 3, under CPL. The second-generation secondary osteon in the center of the image is flattened and appears elliptical rather than circular in the cross-section; (**E**) Some longitudinally oriented secondary osteons in slide 2 (imaged under CPL) anastomose radially, sometimes serially connecting more than 2 osteons and resembling radial vascularization. These are in line and parallel with each other, oriented obliquely across growth zones from the medullary cavity towards the periosteal margin (to the bottom of the image); (**F**) Primary vascular canals (red arrow), primary osteons (purple arrows), and secondary osteons (black arrow) in the cortex of slide 2 under CPL. The developing secondary osteons from erosion lacunae are also noteworthy (white arrow). All scale bars are 500 µm.

Proceeding periosteally, the inner cortex has a few large and some small- to medium-sized erosion lacunae, many developing into large irregularly shaped secondary osteons (as evidenced by thin lamellae marking their perimeter, Figure 4F). The bone is lamellar-zonal with mostly longitudinally arranged vascular canals, with most arranged in circular rows (Figure 4D,E and Figure 5A), but simple primary radial (Volkman’s) canals are also present, and become more abundant in the outer cortex (Figure 5B).

The bone is well vascularized. Longitudinally oriented secondary osteons (some of second, and a few even third generation, Figure 5C,D) dominate. As other vascular canals, most are arranged in circular rows, but several anastomose radially, and most of the latter do it obliquely from the position of the medullary cavity towards the periosteal surface (Figure 5E). Overall, most secondary osteons are circular in shape in the cross-section, but some are flattened and appear elliptical. The orientation of the latter varies greatly, but the long axis is often aligned with the lamellae of the periosteal bone (Figure 5D,E). Sometimes it is perpendicular, and other times at an angle, however. Primary osteons and some longitudinally oriented primary vascular canals also exist and are fairly numerous (Figure 4E and Figure 5F). Osteons and other longitudinal vascularization are greatest in the inner or deep cortex, and steadily diminish periosteally.

**Figure 6:**
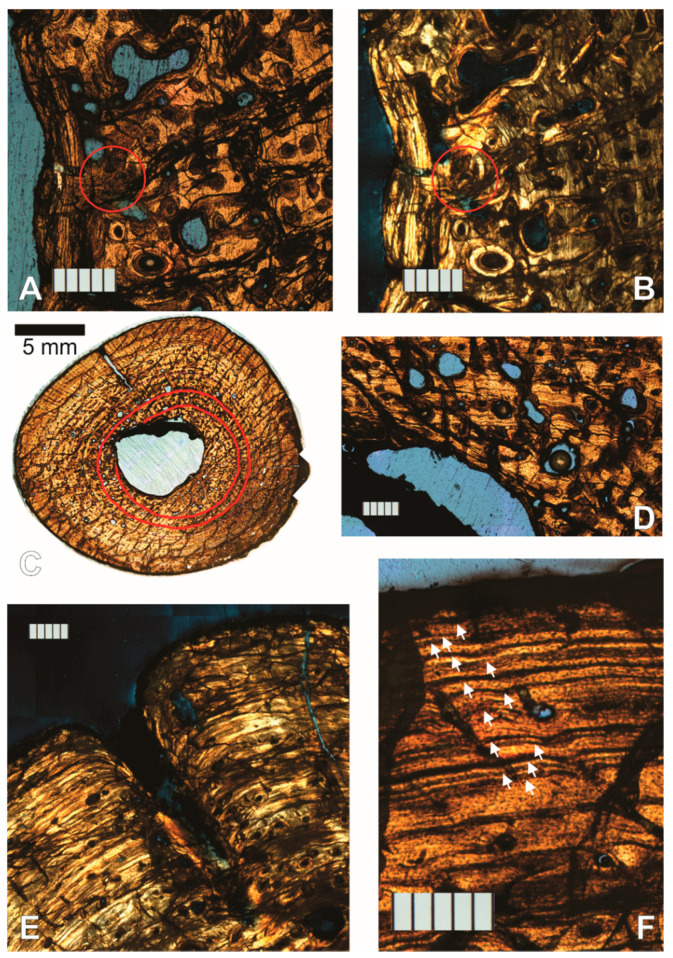
Additional images of thin sections of the left humerus of NJSM 23368, *Hyposaurus rogersii*. All scale bars are 500 µm, except in C, where it is 5 mm. (**A**) Posterolateral endosteal margin of slide 2 under ordinary transmitted light; (**B**) Posterolateral endosteal margin of slide 2 under CPL. The area circled has remnants of previous generations of osteons in interosteonal spaces. Secondary osteons are large and abundant, and the endosteal lamellae cut into the deep periosteal deposits, evidencing drift of the cavity; (**C**) Slide 3 under ordinary transmitted light, with two lines of arrested growth (LAGs) near the medullary cavity highlighted by a thick red line, showing the medullary cavity to be off-center, as it cuts into deposits that once contained part of the innermost marked-up cyclical growth mark (CGM). Top is anterior, right is lateral; (**D**) Endosteal lamellae clearly cut into the inner periosteal lamellae at an angle in this image from the deep anterolateral quadrant of slide 1, evidencing the growth of the medullary cavity and resorption of the deep periosteal cortical deposits. Taken under ordinary transmitted light; (**E**) Mid-diaphyseal nutrient foramen in slide 2 under CPL. Lamellae clearly bend inward along its periphery, including those at the periosteal surface. Because it is longitudinally obliquely oriented and the section is transverse, it does not appear to go through to the medullary cavity on the depicted plane. See Figure 3 for the external position of this foramen on the bone surface, in anterior view; (**F**) External fundamental system (EFS) in the left humerus of NJSM 23368, slide 1 under ordinary transmitted light. At least 13 closely spaced LAGs (arrows) follow the last zone centrifugally, towards the periosteal surface.

Although no reversal line is clear, there are signs of moderate drifting of the medullary cavity. Between the lamellar endosteal layer lining the cavity and the lamellae of the cortex, on the posterolateral side of the cavity, there is a small transitional zone with indications of remodeling: the orientation of the lamellae is not parallel to those above it, and varies greatly, secondary osteons are large and abundant, multiple generations of them are present, and fragments of previous osteon lamellae sometimes occupy inter-osteonal spaces (Figure 6A,B). Additionally, by slide 3, the circumference of the growth marks in the section is very clearly not at the center of the cavity (Figure 6C). The endosteal lamellae meet the deep periosteal cortex lamellae at an angle on the anterolateral side of slide 1 (Figure 6D), indicating endosteal deposition as the cavity drifted.

Osteocyte lacunae are present and fairly abundant throughout the entire sample. Their long axis varies in orientation but is generally oriented in the direction of the lamellae that contains them, which is also the case inside osteonal lamellae (Figure 4D,E).

The preservation of each lamella in most sections of the left humerus is so exceptional that it is difficult to discern lines of arrested growth (LAGs), but growth cycles clearly exist. Because the different sections were finished to different thicknesses for contrast, and due to cracks in the specimens (which could be either a result of slide preparation or preexisting cracks), and preservation differences, it is not possible to trace every growth mark completely around in all sections, and thus counts do not always match exactly between sections. Nonetheless, at least nine clear cycles are visible in all left humerus sections. Growth marks are clearest in section 3. Lamellar thicknesses within the innermost cycle are much larger than in the rest of the bone section. A large mid-diaphyseal nutrient foramen appears in the outer third of the periosteal cortex. Lamellae clearly bend inward along its periphery, including those at the periosteal surface (Figure 6E). It does not appear to go through the cortex into the medullary cavity because nutrient foramina are longitudinally obliquely oriented, and the transverse cross-section plane intersects it at an angle.

Near the periosteal surface, there is an external fundamental system (EFS, Figure 6F). Previously reported by Riqles et al. in Triassic ancestral crocodylian pseudosuchians [12], Klein et al. and Woodward et al. in the extant American alligator [13,14], and by Andrade and Sayão in the much more closely related *Guarinisuchus munizi* [5], this microstructure signifies that skeletal maturity was attained by NJSM 23368, and further supports determinate growth in dyrosaurid crocodylomorphs. Not only are there LAGs stacked up closely at the periosteal surface, but the distance between annual CGMs near the periosteal surface decreases markedly and rather drastically for zonal tissue (measurements provided in Table 1 are from the right humerus but are representative of the left). Furthermore, vascularization in the humerus of NJSM 23368 diminishes significantly and rather suddenly in this area, typically avascular or poorly vascularized in an EFS [14].

At the periosteal surface, there is a diagenetically altered layer of bone that appears dark under transmitted and polarized light. It obscures the histology almost completely, but thin lines and occasionally small irregularly shaped vacuities are barely visible through it in segments of other limb bone sections (see the left femur description below). This dark outer layer of altered periosteal cortex was previously reported by Boles in much of the vertebrate material he sectioned, which originated from the Inversand site [15].

#### 3.1.2. Right Humerus

Two thin sections were made from the right humerus sample, labeled in order from proximal to distal. As in the case of the left humerus sections (and as expected), the histology is essentially the same among the right sections. It is also very similar to that of the left humerus sections, and the EFS is also present. A notable difference between sections 1 and 2 is that 2 has more and larger erosion bays surrounding the medullary cavity, and also has more erosion cavities in the outer cortex than 1, although they are generally only slightly larger there.

**Figure 7:**
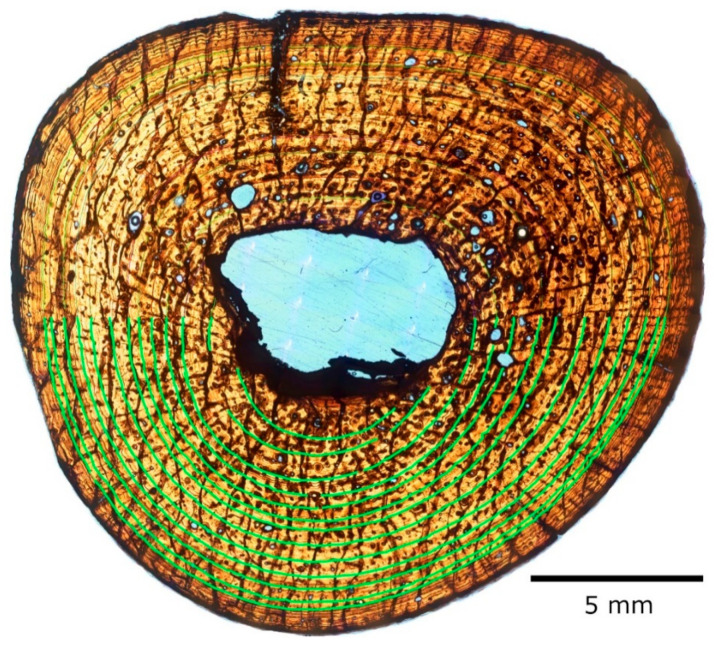
Right humerus of NJSM 23368, mid-diaphyseal transverse section, slide 1 under ordinary transmitted light. Thirteen CGMs are visible, enhanced for illustration here in the bottom (posterior) half of the image by tracing with green lines (brush size 50 pixels, hardness 100). The top (anterior) half has lines as traced for study (different colors per line, brush size 20 and hardness 50, which results in 10-pixel-wide lines at their core). Scale bar is 5 mm.

Thirteen cycles are visible in section 1, of which the innermost two are only half present, having been partially engulfed by the growing and drifting medullary cavity (Figure 7). The fine endosteal lamellar layer that surrounds the medullary cavity in most sections is present only partially, obscured by a dark band of similar appearance to the strut that divides the cavity, and seemingly partially resorbed.

Because the preservation of cycles is better in the right humerus than in all other sections, the skeletochronological analysis will be centered on it.

#### 3.1.3. Left Femur

**Figure 8:**
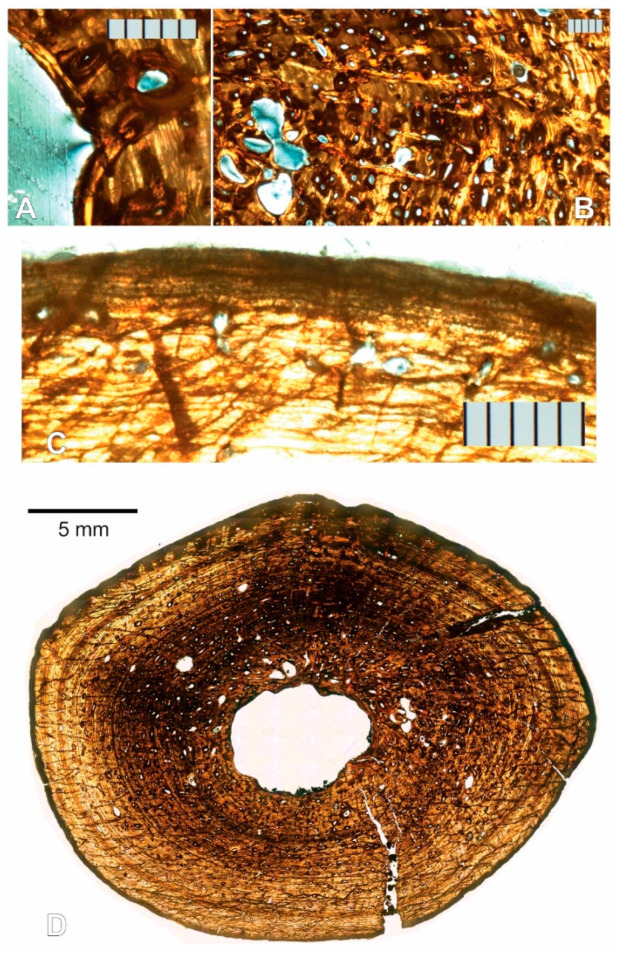
Mid-diaphyseal thin sections of the left femur of NJSM 23368, *Hyposaurus rogersii*. All scale bars are 500 µm, except in D, where it is 5 mm; (**A**) Endosteal lamellae cutting into the deep periosteal deposits in slide 2 under CPL. Secondary osteons abound in the periosteal deposits; (**B**) Radial anastomoses between longitudinal osteons and large erosion lacunae in the inner periosteal cortex of slide 1 under CPL; (**C**) Segment along the periosteal perimeter of slide 2, which under CPL reveals some of the histology normally obscured by a dark diagenetic band present in most sections from the Inversand site. Several closely stacked LAGs that could be an EFS are present, as are a few irregularly shaped vacuities and small circular vacuities, presumably primitive vascular canals; (**D**) Slide 1 under ordinary transmitted light. Top of the section image is anterior, left is medial, right is lateral, and bottom is posterior.

Two left femur thin sections were made and labeled sequentially from proximal to distal. While the histology is similar to the humerus’, there are some differences. The medullary cavity is completely open (there is nothing dividing it), and it is smaller in the femur than the humerus, occupying roughly 7% of the cross-sectional area in the left femur while it occupies roughly 9% in each humerus (including the strut; see Supplementary Materials Table S1). The endosteal lamellae clearly cut into the periosteal deep cortex (Figure 8A), showing clear erosion of the periosteal cortex, and growth or drift of the medullary cavity. In section 1, the large erosion bays appear mostly on the anterior half of the section (Figure 8D). The left femur shows a higher amount of remodeling than either of the humeri, evidenced by the larger number of erosion bays, and their overall larger size. Radial anastomoses between longitudinal osteons exist throughout the cortex but are most common and longer in the inner half (Figure 8B). These often occur serially, at times joining three or more longitudinal osteons periosteally, resulting in a pattern reminiscent of radial vascular canals. Although the longitudinal osteons are arranged in circular rows, they also often almost line up as if in radial rows. Vascularization in general diminishes periosteally, as in the humeri. Not unexpectedly, this may indicate a changing growth rate through the specimen’s life history, it being faster early on.

**Figure 9:**
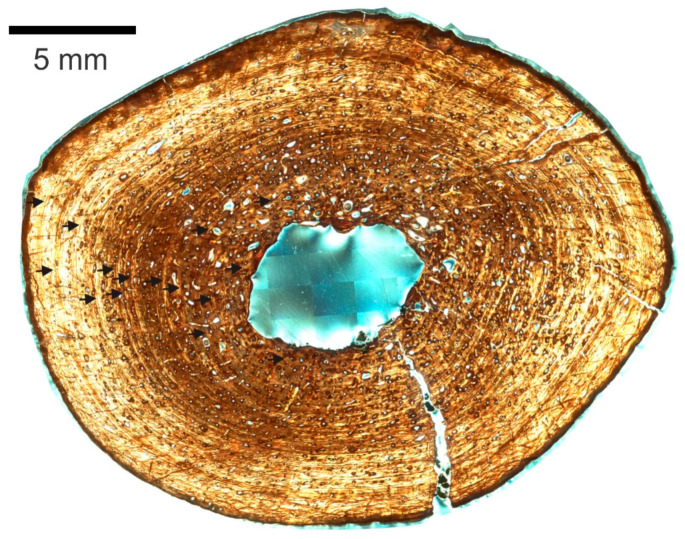
Mid-diaphyseal transverse thin section of the left femur of NJSM 23368, *H. rogersii*, slide 2 under CPL. Fifteen growth cycles are visible (arrows); however, they are variably preserved throughout the section, and several cannot be traced completely around. Scale bar is 5 mm.

Fifteen growth cycles are present; however, they are variably preserved throughout the section and several cannot be traced completely around (Figure 8D). They are most clear under CPL on slide 2 (Figure 9). Section 2 also has a segment along its perimeter that under CPL reveals some of the histology normally obscured by a dark diagenetic band, present in most sections from the Inversand site (Figure 8C). Revealed are several closely stacked LAGs, a few irregularly shaped vacuities, and small circular vacuities, presumably primitive vascular canals. These could simply be part of the repeating cycles of stacked LAGs between wider zones present in the rest of the cortex, but the decrease in vascularization toward this microstructure supports it being an EFS as in the humeri. While it is certainly possible for one element in a skeleton not to possess an EFS when others do (for example, see the pedal phalanx contrasted to other elements of *Hypacrosaurus stebingeri* reported by Horner et al. [16]), it is unlikely that this element would have grown significantly larger had the specimen not died.

#### 3.1.4. Right Femur

**Figure 10:**
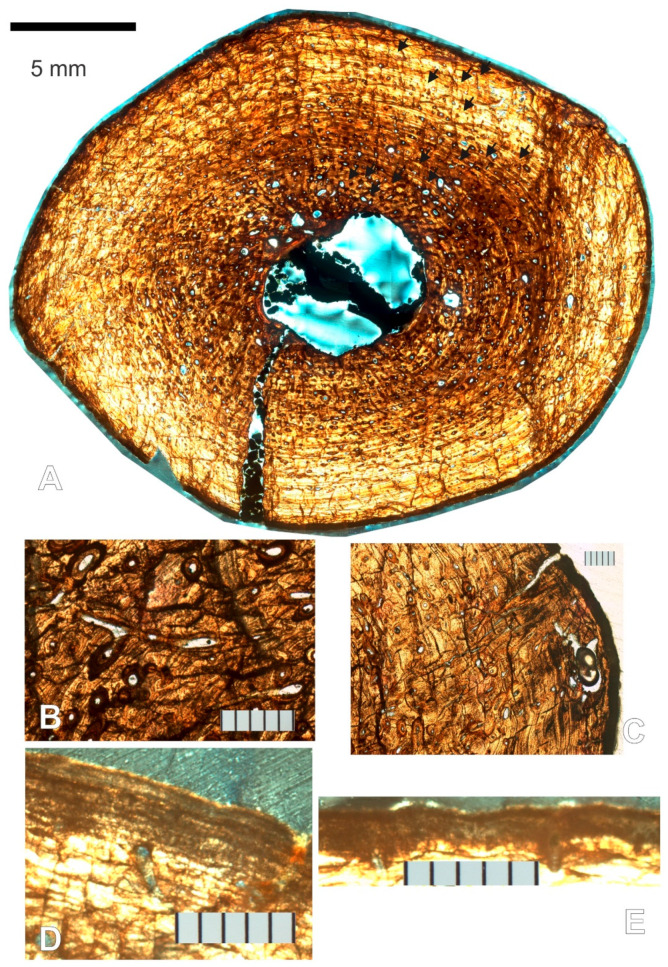
Mid-diaphyseal transverse thin section of the right femur of NJSM 23368, *H. rogersii*. All scale bars except for that in A are 500 µm; (**A**) Slide 1 under CPL. Top of the image is anterior, right is medial. Glauconite is visible filling a crack that leads to the medullary cavity, where a few grains are present. The arrows point to the 14 visible CGMs. Scale bar is 5 mm; (**B**) Secondary osteons with reticular anastomoses in slide 2 under ordinary transmitted light; (**C**) Sharpey’s fibers are present through the periosteal surface of slide 2, photographed under ordinary transmitted light; (**D**) Closely stacked LAGs suggestive of an EFS can be seen through the obscure diagenetic band at the periosteal surface of slide 2 under CPL; (**E**) Other segments of the perimeter of slide 2 (even under CPL as shown) reveal only dark irregularly shaped vacuities that may represent Wedl tunnels.

There are two right femur thin sections, made and labeled sequentially from proximal to distal as in the other elements. The histology, as expected, is essentially the same as described for the left femur, except the structure in the medullary cavity is present as in the humeri. The cavity occupies roughly 6% of the cross-sectional area if the strut is included (Supplementary Materials Table S1). Glauconite grains from the sedimentary matrix are also present within the medullary cavity, where they presumably entered through an open crack on the bone that reaches inward from the cortical surface, and that is partially filled with glauconite itself (Figure 10A).

As stated for the left femur, the level of remodeling is higher in the femoral than in the humeral sections. This is further evidenced by a higher concentration of longitudinal secondary osteons, more of which are joined not only by radial anastomoses, but in some instances by reticular anastomoses (Figure 10B).

Sharpey’s fibers are visible in section 2 near the cortical surface (Figure 10C). This section also presents a murky view of the dark band that obscures the periosteal surface. In some segments, only dark irregularly shaped vacuities are visible within (Figure 10E), but in others under CPL light, closely stacked LAGs can be seen, suggestive of an EFS as in the left femur (Figure 10D). The irregularly shaped vacuities are not clear enough to identify confidently, but Boles reported Wedl tunnels (micro tunnels that represent microbial bioerosion during bone diagenesis) in similarly altered bone from the same site [15].

Fourteen growth cycles are visible in these sections (Figure 10A), and the CGMs are clearest in the medial half of the section (preservation issues obscure most of the cycles in the lateral half, which corresponds to the left side of Figure 10A). The inner CGMs are in most instances untraceable in the inferior end of the anterolateral quadrant, and the superior end of the posterolateral quadrant. They are obscured by secondary osteons and erosion bays presumably related to the expansion and drift of the medullary cavity, which appears to have moved posteromedially.

### 3.2. Skeletochronology

The following thin sections were studied in detail under transmitted light and circularly polarized light (CPL), then measured: left humerus 3, right humerus 1, right femur 1, and left femur 2. Firstly, major and minor axes were identified, and then cyclical growth marks were identified and traced as far as preservation allowed. The distance from the centroid (as determined by the intersection of the major and minor axes, following Horner and Padian and Woodward et al. [17,18]) was measured to the first preserved CGM, and from there on to the next, successively, until the last preserved CGM was reached (progressing centrifugally, along both the major and minor axes, and when preservation allowed on both sides of the centroid). The measurements were taken in pixels as described in the Materials and Methods, and entered into a spreadsheet program (Microsoft Excel 2000), where they were converted to micrometers and processed to calculate intervals, circumferences, means, percentage increases, mean percentage increases, and all other calculations herein.

One slide of each sectioned element was measured and processed in a similar manner. We assumed the CGMs in our samples are annual (see Discussion). As expected, left and right element pairs produced similar results [19], with some minor discrepancies attributable to differential preservation. Despite cracks in the section, the right humerus slide no. 1 preserved the most CGMs that were traceable completely around the circumference without other artifacts of preservation or preparation obscuring at least part of the CGMs. To ensure accuracy and precision as much as possible, this report will focus on the results obtained from the major axis of right humerus number 1 (Figure 7, Table 1). Results from right femur slide 1 will also be discussed for comparison, but it must be noted that they were obtained from a single quadrant of the slide due to poor preservation of CGMs along the axis on the respective opposite side of the centroid (Figure 10A, Table 2).

Rather than run all the retrocalculation models using the distance to the centroid of the bone from the first fully preserved CGM, we decided to use the distance from the estimated cortical surface of a neonate element to the first fully preserved CGM. We reasoned that a neonate element would not contain an annual CGM, and could stand in for the size of the original medullary cavity before any expansion partially destroyed the growth record, as Pellegrini did with the mosasaurid *Clidastes* [20]. Because we do not have a neonate fossil *Hyposaurus rogersii* to section or even measure, and because all crocodilians hatch at similar sizes [21], we measured and used an extant *Alligator mississippiensis* hatchling (MOR-OST 1647). Its left humerus and right femur were previously sectioned transversely at the midshaft and imaged by the Museum of the Rockies, which kindly allowed their use for our research. This specimen served as a neonate stand-in for the retrocalculation of CGMs of the respective *H. rogersii* elements, both left and right.

Following Horner and Padian and Woodward et al. [17,18], we retrocalculated missing CGMs using five different models: (1) dividing the distance from the neonate periosteal surface to the first CGM by the broadest CGM interval in the section; (2) dividing said distance by the length of the penultimate interval preserved; (3) dividing it by a mean interval instead; (4) using a mean percentage centripetal increase factor applied to the first preserved interval to calculate the first missing CGM interval, and then on to that result to calculate the second, and so on, until the neonate periosteal surface is reached; and (5) a parabolic model in which the interval lengths of each successive interval past and before the first preserved interval mirror each other.

#### 3.2.1. Right Humerus

Using the broadest interval band as a denominator, the section to the left of the centroid produces an estimate of 3 missing years, which makes the age of the specimen 15 after accounting for the innermost CGM (thrown out of the calculations due to medullary cavity expansion along the major axis obliterating the intersection point; see paragraph on the mean percentage increase model for details). The section to the right of the centroid produced an estimate of 4.5, which is congruent as it preserves only 11 CGMs, and thus totals 15 to 16 years of biological age (Supplementary Materials Table S2).

When the penultimate interval is used instead, the results are respectively 6.5 and 10.6 (Supplementary Materials Table S2), which mean 18 to 19 and 21 to 22 years of age. This model presents the biggest disagreement between the left and right of the section. Even if we select the ends of the ranges that are closest to each other, the left and right section results are off by 2 years. The age estimate is considerably higher than in the other models, as well.

The mean interval produces results of 5.6 and 6.9 (Supplementary Materials Table S2), which mean 17 to 18 and 18 years of age for the specimen, respectively.

Although the growth lines are generally regularly spaced, the interval distances between them vary, not always decreasing centrifugally (Table 1). This may seem strange or unexpected but has been reported before in other groups like dinosaurs [16,22]. This results in annual percentage decreases mixed in with annual percentage increases, and makes any results obtained by a centripetal mean percentage increase retrocalculation model for CGMs questionable (Supplementary Materials Tables S3 and S4). Nonetheless, this model produced an estimated two to three (to the left of the section) or three to four (to the right of the section) retrocalculated LAGs along the major axis. The section to the left of the centroid preserves 12 LAGs that intercept the major axis, but there is another LAG that is cut off by the expanding medullary cavity a short distance from the axis, which would reduce the estimate of missing LAGs to 2 (or less) and estimate the total LAGs in the section (and the biological age of the specimen in years) at 14. The section to the right of the centroid only preserves 11 LAGs, which is congruent with the previous result of 14 after adding the 3 (to 4) retrocalculated LAGs.

The parabolic model allows for five to six missing CGMs to the left of the centroid, and six to its right. This would mean the specimen’s biological age was 17 to 18 years (Supplementary Materials Table S5).

#### 3.2.2. Right Femur

Using the broadest interval, the section to the right of the centroid produces an estimated 3 years missing from the record (Supplementary Materials Table S6), which makes the specimen 16 years old at the time of death. As in the right humerus, this accounts for the first CGM present in the minor axis not being preserved along the major axis due to endosteal resorption.

The penultimate-interval-as-denominator model produces a drastically different estimate: 13 missing CGMs (Supplementary Materials Table S6). This would make the specimen 26 years of age at death.

The mean interval model estimates 6 missing cycles (Supplementary Materials Table S6), for a total age at death of 19 years.

As in the humerus, the intervals decrease and increase centrifugally (Table 2). This results in annual percentage increases and decreases, which make the accuracy of the centripetal mean percentage increase retrocalculation model questionable for our specimen. It estimates 5 CGMs are missing (Supplementary Materials Tables S7 and S8), putting the specimen at an age of 18 years.

The parabolic model also produces an estimate of 5 missing cycles (Supplementary Materials Table S9), making the specimen 18 years old as well.

## 4. Discussion

We interpret the growth cycles as annual. These are cyclical growth marks (CGMs, LAGs, or annuli that together with a zone represent one annual cycle), many traceable through the entire circumference in most sections. They also have a regular appearance and are regularly spaced in general. Non-cyclical marks often appear haphazard rather than regularly spaced and tend to be locally confined to an arc rather than being traceable around the circumference of the transverse bone cross-section [18]. Many authors have addressed the annual periodicity of CGMs. Peabody was among the earliest to do so in reptiles [23], Castanet and Smirina reported on amphibians in addition to reptiles [24], and Castanet et al. provided a good general overview of similar work in many tetrapod groups [25]. Woodward et al. provide additional references, including further work on groups other than reptiles [18]. However, specifically within extant crocodiles, the annual nature of CGMs was tested with positive results by Buffrénil [26], Hutton [27], Tucker [28], and Woodward et al. [19]. Our interpretation of CGMs being annually formed in our specimen is based on their work. This is in agreement with Andrade and Sayão, who inferred the growth cycles in their dyrosaurid material were annually deposited as well [5].

Sectioning the paired humeri and femora of NJSM 23368 allowed the benefit of compensating for any obscured histology due to differences in preservation within the pair. Woodward et al. found that in the alligators they sampled, there was essentially no difference in the diaphyseal circumference or CGM circumference between the left and right elements of an individual, if sampled roughly in the same diaphyseal location [19]. Only our left humerus provided different CGM counts within its serial sections (four were made, the most of all elements sampled herein). This would appear contrary to Woodward et al. [19], whose results indicated that although medullary expansion rates and secondary remodeling may affect counts between elements of an individual, serial diaphyseal thin sections of the same bone produce the same CGM counts. However, the histological structures observable in our fossil material sometimes varied between serial sections, due to both preservation variances and, more so, to preparation accidents like sample loss or having ground a section too thin (thus losing contrast). This sometimes created an inability to trace the CGMs through the circumference. When this happened, we made more sections (hence four for the left humerus rather than two as in the other elements). While the left humerus sections have 9 CGMs, the right have 13, and the left femora 15, while the right 14 (having partly lost the innermost CGM present in its counterpart to preservation issues).

In order to retrocalculate missing CGMs lost to resorption, we determined the centroid of the section and measured the distance from it to each preserved CGM, and subtracted the distance from the centroid to the cortical surface of a hatchling. No *Hyposaurus* hatchling humeri or femora are known, but extant crocodylian neonates are all approximately the same size: 20–30 cm total body length (TL), 30–100 g [21]. This is the case across the entire order, and thus it is reasonable to expect this holds true for extinct forms and their relatives, like *Hyposaurus*. Therefore, it is also reasonable to assume the mid-diaphyseal thickness of the femora and humeri of extant hatchling crocodylians is comparable to that of *Hyposaurus* hatchlings. In their study of intraskeletal histovariability in *Alligator mississippiensis*, Woodward et al. used previously prepared mid-diaphyseal thin sections of an alligator hatchling skeleton, MOR-OST 1647, which included a right femur and left humerus [19]. Their midshaft transverse cross-sectional radii are 0.6636 mm for radius a and 0.6324 mm for radius b of the femur, and 0.7076 and 0.5580 mm respectively for the humerus. We obtained these measurements directly for our study from high-definition images of the slides and their scan settings, generously provided to us by the Museum of the Rockies. Then, we ran our measurements through five different models to retrocalculate missing LAGs, following Woodward et al. [18], and obtained the following results:

(1) If using the thickest band for retrocalculation, the humerus indicates the specimen would have been 15 to 16 years old (and growing, as in each model) at the time of death. The femur results are congruent.
(2) If using the penultimate band in the humerus, it would have been 18 to 22. The femur, in contrast, estimates 26.
(3) If using a mean band thickness, the humerus suggests it would have been 17 to 18. The femur data suggest 19.
(4) If using a mean percentage increase, the humerus results in an estimate of 14 to 15, while the femur estimates 18.
(5) If using a parabolic model, the humerus indicates the specimen would have been 17 to 18, and the femur 18.

Of the above, model 4 is probably least reliable in our sections, as the thickness of the growth mark bands varied rather than always decreased over time. The penultimate band (used for model 2) is actually relatively thick, thicker than the band that precedes it, and thicker than the last band. The left and right sides of the right humerus and femur sections studied also produce the most different results between them (compared to results from the left and right in other models), so it lacks accuracy and also precision: the result range is much wider than in the other models. This leaves models 1 (which likely underestimates age, as it uses the highest growth rate all throughout), 3, and 5, the latter two of which generally agree with each other. Therefore, we estimate NJSM 23368 was 18 years old, give or take one year, at death.

*Hyposaurus rogersii* did not reach the size of the Nile crocodile (*Crocodylus niloticus*), or the salt-water crocodile (*C. porosus*); its adult total body length (TL) has been estimated at 2.48 to 3.11 m [29] and at 3 to 3.5 m [30]. Furthermore, based on the body/skull chart proportions in Jouve et al. [30], Callahan et al. estimated a total body length of 2.8 m for our specimen, NJSM 23368 [31]. However, *H. rogersii* was a marine crocodylian like *C. porosus*. While the American crocodile (*C. acutus*) is not quite as marine as *C. porosus*, we believe it is the best extant analogue to *H. rogersii* in terms of relative size (typically 2.3 to 3.7 m TL [32]), and marine capacity, even if not in diet. Richards observed five life history stages in the American crocodile: hatchling, juvenile, subadult, young adult, and adult [33]. Briggs-Gonzales et al. recognized essentially the same stages, except for lumping young adults and adults into the adult stage [34]. Sexual maturity (or breeding age) in *C. acutus* is reached at 2.25 m or longer total length (TL) [34]. This corresponds to the young adult stage of Richards [33], where TL is 2.25 to 2.5 m, a stage added because smaller females lay a smaller clutch of eggs than females longer than 2.5 m TL [35]. Briggs-Gonzales et al. estimated 2.25 m TL was reached by more than 5% of the population of females in Southern Florida at 8 years of age, with an estimated 2% reproducing at age 7 [34]. Richards had estimated breeding age at 10 [33], while Moler had it at 9 (also in Florida) [36], and Thorbjarnarson at 10 for populations in Haiti and Jamaica [37]. Ferguson provides comparative data for other extant crocodilians; all except for some populations of the Nile crocodile (*C. niloticus*) reach sexual maturity by age 18 [38]. Given the estimated age from skeletochronology (18 years) and estimated length (2.8 m), NJSM 23368 was an adult, contrary to the determination of subadult by Callahan et al. [31]. Moreover, the presence of an EFS—a bone microstructure that indicates the effective cessation of periosteal growth [14]—in both humeri, and its likely presence in both femora proves NJSM 23368 was skeletally a fully grown adult. The external morphology supports this, as the specimen exhibits fully fused neurocentral suture closure along the spinal column, indicating maturity [39]. This is a bit unexpected because larger and more robust (even if less complete) specimens of *Hyposaurus* are known, among them NJSM 11069 and 12293, and Yale Peabody Museum (YPM) 985 and 753 (Table 3). Both of the NJSM specimens are from the same locality as NJSM 23368, and although one was collected from the Upper Hornerstown Formation, like NJSM 23368, the other was derived from the basal Hornerstown Formation. This rules out a general reduction in size for the species as time progressed. A more likely possibility is sexual size dimorphism, as in all extant crocodylians like the American [40], Nile [41], and especially the saltwater crocodile [42], where the males are larger than the females. Unfortunately, examination of the extent of bone remodeling and porosity in the cortex of the femora, and comparison to that of published femoral midshaft cross-sections of extant, egg-laying, and other alligators in studies by Wink and Elsey [43] and Klein et al. [13] does not conclusively allow the determination of the sex of our specimen. Although the overall modest amount of bone porosity in the humeri and femora of NJSM 23368 does not suggest that it was egg-laying at the time of death, Wink et al. stated that femoral density for mature alligators returned to normal levels 1–2 months after egg-laying [44]. The non-egg-laying female alligator section in Figure 2 of Wink and Elsey [43] is grossly comparable in porosity to the femoral sections of NJSM 23368. In fact, the latter’s porosity may be lesser than the former’s, indicating it was either a non-egg-laying female or a male. The extent of remodeling does not aid in resolving between the two: although there are abundant secondary osteons (especially in the femora), most are first-generation and regularly shaped longitudinal secondary osteons. Erosion bays are few and most are modest in size, rarely occurring far from the endosteal margin. It is necessary to increase the individual sample size of *Hyposaurus rogersii* femoral midshaft cross-sections in order to establish a baseline for comparisons in cortex porosity and remodeling extent, and to quantify results and compare them further to the results in Wink et al. for extant alligator femoral robusticity [44], to confidently infer the sex of the specimens from this index. We intend to further research the matter and publish results in a future work. At least until then, sexual dimorphism is the likeliest explanation for the difference in overall size between our fully grown specimen and the known larger more robust specimens of *H. rogersii* in various museum collections, suggesting therefore that NJSM 23368 was a female.

The histology of the midshaft of the bones examined shows no signs of the specializations in bone microstructure typical of tetrapods secondarily adapted to an aquatic lifestyle (summarized by Ricqles and Buffrénil [45]). The open medullary cavity and lack of trabeculae completely dismiss the possibility of an osteoporotic-like state. The thin endosteal layer surrounding the medullary cavity is distinct from the periosteal layers centrifugal to it, ruling out an osteosclerotic state by endosteal filling of the medullary cavity. Haversian reconstruction is not extensive enough to suggest an osteosclerotic state by itself, and there is no indication of inhibition of normal chondroclastic and osteoclastic activities in the samples to cause an osteosclerotic state, either: the open medullary cavity does not support it, and there is no calcified cartilage in the diaphysis. External morphology of the elements studied before sectioning, which are generally comparable to those of extant crocodiles and slender by comparison to more robust specimens of *Hyposaurus*, reveals no indication of pachyostosis by hyperplasy of the periosteal cortex (Figure 1). The histology of the cortex confirms this (Figure 4A, Figure 6C, Figure 7, Figure 8C, Figure 9 and Figure 10A). Given the marine sediments NJSM 23368 was recovered from, the morphology of its skeleton, and the reported histology of the South American dyrosaurid *Guarinisuchus munizi* by Andrade and Sayão [5], these results are puzzling. All subsequent literature on *Hyposaurus* agrees that it was a marine swimmer as established by Troxell [46]. The sediments the adult individuals of the species have been recovered from are certainly marine; for example, see [47] (p. 7) for associated fauna of the Inversand site, all marine. The narrow snout with close-set homodont teeth indicates a piscivorous diet [48], and multiple other characters in the skeleton evidence adaptation to an aquatic environment (described by Troxell [46] and summarized by Denton et al. [3]). Andrade and Sayão sectioned a femur of *G. munizi* within the diaphysis, but because only the proximal third of the element was preserved, not exactly at the midpoint [5]. They encountered a very thin cortex and extensive trabeculae that filled most of the medullary cavity, despite being close to the mid-diaphysis. The trabeculae extended throughout the area normally filled by compact bone, left a clear medullary cavity, and had originated through remodeling and resorption of primary tissues. They concluded this was consistent with osteoporosis and a fast-swimming ecology. They also noted some lamellar thickening of the trabeculae, which they interpreted as consistent with minimal osteosclerosis, which is also associated with a fully aquatic lifestyle and compatible with a fast-swimming ecology. Therefore, they suggested a semi-aquatic marine lifestyle and fast-swimming ecology for *G. munizi*, although lacking specialized adaptations for a fully marine life. We found no evidence of any of these microstructural adaptations in NJSM 23368 (probably because we were able to sample exactly at the mid-diaphysis), which supports a more traditional interpretation. Langston reported that the angulation of the zygapophyseal facets of the anterior thoracic and posterior cervical vertebrae of hyposaurines was so high that the trunk was stiff and capable of limited lateral flexion only [48]. Therefore, he concluded swimming must have been achieved chiefly by undulations of the tail. Movement of the limbs perhaps assisted this, as Denton et al. later remarked [3]. Schwarz-Wings et al. further stated that “For hyposaurine dyrosaurids, muscle reconstructions of the tail, sacral and lumbar region with tall cross sections and long muscle fibers indicate a higher frequency of lateral undulation and a more powerful movement of the tail than in extant crocodylians” and “the lateral surface of the tail being two-thirds as large as in extant crocodylians … hyposaurine dyrosaurids were surely capable of producing considerably more thrust than extant crocodylians. The amount of lateral flexibility of the lumbocaudal region of hyposaurine dyrosaurids allowed high amplitude undulation. The terminal half of the tail could be used for steering as in extant crocodylians (Frey and Salisbury, 2001 [49]; Salisbury and Frey, 2001 [50]). As reconstructed, the axial swimming capabilities of hyposaurine dyrosaurids exceed those of extant crocodylians with respect to velocity, stamina, acceleration and maneuverability, which was improved by the high flexibility of the tail base” [51]. The above strongly suggests that *H. rogersii* was a swimmer in the ocean and swam faster and better than modern crocodilians, but it was not a fast swimmer in the sense of cetaceans or ichthyosaurs, for example.

The lack of bone microstructure specializations typical of tetrapods secondarily adapted to an aquatic lifestyle in the limb bones of *Hyposaurus* is as strange as the limb proportions in all dyrosaurids. Swinton describes these in *Congosaurus*, noting the humerus is longer than any bone except the femur, barely 5 mm longer, and notes that in *C. acutus*, the humerus is much shorter than the femur [52]. Langston further states that in amphibious crocodilians and in marine mesosuchians, the forelimbs are significantly shorter than the hindlimbs, but in dyrosaurids, they are nearly the same size and then remarks that the functional significance of the relatively powerful forelimbs is not clear [48]. Schwarz-Wings et al. present some thoughts on that, pointing out that although dyrosaurids inhabited marine, epicontinental, and coastal environments, they have also been recovered from brackish, estuarine, and fluviatile environments, and with their strong limbs, hyposaurine dyrosaurids could brace themselves in gullies with rapid water flow and probably crawl against currents [51]. They further propose that juvenile hyposaurines (capable of high-walk and gallop based on their reconstruction) could plausibly have covered large distances on beaches and riverbanks. These interpretations would explain why the forelimbs were never reduced and why they lack specialized adaptations for a fully marine life yet were still successful shallow marine predators. Further insight is provided by consideration of the gastroliths recovered with NJSM 12293, another *H. rogersii* excavated at the Inversand site [3]. Based on comparative analysis of gastrolith distribution, Taylor suggests that secondarily adapted aquatic animals with gastroliths are at least sometimes more dependent on relative maneuverability and slow swimming, than on fast-swimming pursuit to catch prey [53]. He further mentions elasmosaurid plesiosaurs and extant crocodilians, ambush predators, make a good example, while penguins and otariids seem contrary exceptions as active pursuit predators. *Hyposaurus rogersii* may have used gastroliths as ballast when diving (a faster and more efficient functional alternative in terms of sinking force to developing pachyostosis [53]), and for buoyancy control in swimming (to prevent tail-heaviness while floating, and to control rolling as proposed for the Nile crocodile by Cott [41]). Computer modeling suggests gastroliths in crocodiles are always too few and light to act as ballast, however [54]. This may be the case, as Uriona and Farmer showed the crocodylian hepatic-piston breathing system and related musculature can shift the relative position of the lungs in alligators, which when floating allows them to control their buoyancy, diving and rolling at will without major exterior movements (gastroliths not required) [55]. Nonetheless, Wings reviewed most publications cited here before 2008 and other evidence, and although considering the hypothesis of controversial status (in contrast to implausible or plausible) concluded that “the issue of hydrostatic function of gastroliths in aquatic vertebrates”, in general, remained unsettled [56]. Even if the position of the lungs is the principal means for buoyancy control, gastroliths may provide a supplemental benefit. Therefore, the gastroliths in *H. rogersii* may be evidence against a fast-swimming ecology. Although the evidence suggests *Hyposaurus* was a faster and better swimmer than any extant crocodile, and although it likely went out farther into the sea than even *C. porosus*, it seems *H. rogersii* was still a shallow marine predator that generally remained close to the coast, as suggested by Hua and De Buffrénil [57]. Despite what may have been formidable swimming capabilities, it is more likely *Hyposaurus* was an aquatic ambush predator like Denton et al. proposed [3], than a pursuit predator like Buffetaut suggested and a fast-swimming ecology would support [5,58].

In their study of the bone histology of the Thalattosuchia, Hua and De Buffrénil remarked that following their extinction, the dyrosaurids evolved into those adaptive zones, adapting similarly in morphology as well as ecology, but with a swimming efficiency theorized to be intermediate between teleosaurids and metriorhynchids [4,57,59]. They furthermore stated that the dyrosaurids exemplified the physiological conservatism they observed in the Thalattosuchia, and that the known skeletal histology was basically identical to that of their specimens [57]. Andrade and Sayão concurred, and further proposed a fast-swimming ecology based mainly on the microanatomic organization of their *G. munizi* sections as compared to that of metriorhychids reported by Hua and De Buffrénil [5,57]. Our work supports these conclusions in regards to physiology (ecto-poikilothermic) but finds the microstructure of the bones of *Hyposaurus* more like that reported for teleosaurids by Hua and De Buffrénil: lacking any peculiar specialization of skeletal microstructure for a marine ecology [57]. We agree with Hua and De Buffrénil in that hyposaurine dyrosaurids were better swimmers than the teleosaurids but not as good as the metriorhynchids: *Hyposaurus* certainly exhibits reduced lightened osteoderms (contrary to teleosaurids) and the anatomy of the tail and its effective function in swimming have already been discussed. However, *Hyposaurus* lacks the bone microstructure developed in metriorhynchids for fast swimming, and also the reversed, heterocercal tail. Although there are hints of a bend, as Langston pointed out: “the wedging of the caudal centra suggests some downward bending of the tail, though nothing approaching the condition in *Geosaurus* should be inferred” [50].

## 5. Conclusions

The bone microstructure and histology of the midshaft transverse cross-sections of the humeri and femora of *Hyposaurus rogersii* NJSM 23368 show none of the adaptations normally found in tetrapods secondarily adapted to an aquatic lifestyle. Although undeniably a marine predator and a good swimmer, it was not a fast swimmer like modern cetaceans or the extinct metriorhychids, mosasaurs and ichthyosaurs. *Hyposaurus rogersii* was most likely an ambush predator that spent most of its adult life at sea but relatively close to shore.

NJSM 23368 was a skeletally mature individual that died at about 18 years of age. Gross comparison to larger more robust specimens from the same locality (and elsewhere) suggests sexual dimorphism for the species as in modern crocodylians, where males are larger. Because NJSM 23368 is one of the more gracile forms, it likely was a female.

## Figures and Tables

**Table 1:** Distance (in microns) from the centroid to each preserved line of arrested growth (LAG) in the right humerus of NJSM 23368 (slide 1, scan 1), in centrifugal order. Transverse diameters were determined by first measuring the widest point through the center of the bone section (establishing diameter a, the horizontal axis), and then identifying the greatest height at 90° to that line (diameter b, the vertical axis). The intersections of these diameters defined the centroid. Radius a+ is the axis to the right of the centroid, a- is to its left, b+ to its top, and b- to its bottom. N/A = Not Applicable; NP = Not Preserved.

LAG No.	Radius a−	Radius a+	Percent Decrease from Previous Interval (a−)	Percent Decrease from Previous Interval (a+)	Radius b−	Radius b+	Percent Decrease from Previous Interval (b−)	Percent Decrease from Previous INTERVAL (b+)	Calculated Circumference (Negative Radii)	Calculated Circumference (Positive Radii)
1	NP	NP	N/A	N/A	3606	NP	N/A	N/A	N/A	N/A
2	3247	NP	N/A	N/A	4088	NP	−54.7	N/A	23,120	N/A
3	4065	4621	39.37	N/A	4834	2903	39.2	N/A	28,009	23,948
4	4561	5278	5.97	25.1	5287	3572	11.4	8.5	30,981	28,062
5	5027	5770	16.85	−64.1	5689	4183	7.1	−22.1	33,698	31,469
6	5415	6578	26.58	43.7	6063	4930	30.7	39.7	36,086	36,340
7	5699	7032	−102.71	−90.4	6322	5381	−85.6	−59.7	37,790	39,172
8	6276	7898	9.60	16.1	6802	6101	3.5	12.0	41,103	44,161
9	6798	8624	15.80	21.6	7266	6735	0.3	27.2	44,195	48,436
10	7237	9194	10.26	6.6	7728	7196	24.0	20.7	47,027	51,681
11	7631	9726	1.63	30.8	8080	7562	−7.7	−7.0	49,368	54,523
12	8019	10,094	46.51	48.3	8458	7953	38.8	65.8	51,774	56,897
13	8226	10,285	N/A	N/A	8690	8087	N/A	N/A	53,155	57,924

**Table 2:** Distance (to the nearest micron) from the centroid to each preserved LAG in the upper right quadrant of the right femur of NJSM 23368 (slide 1, scan 1 CPL), in centrifugal order. CGM = Cyclical Growth Mark.

LAG No.	Radius a+	Interval between CGMs (Radius a+)	Percent Decrease from Previous Interval (a+)	Radius b+	Interval between CGMs (Radius b+)	Percent Decrease from Previous Interval (b+)	Calculated circumference (Positive Radii)
1	NP	N/A	N/A	3395	407	N/A	N/A
2	4719	412	N/A	3802	283	30.4	26,848
3	5131	263	36.2	4086	258	9.1	29,049
4	5394	1252	−376.5	4343	726	−182.0	30,680
5	6646	773	38.3	5070	438	39.7	36,973
6	7419	1242	−60.7	5507	572	−30.6	40,832
7	8661	592	52.3	6079	510	10.8	46,662
8	9253	922	−55.7	6589	670	−31.3	50,123
9	10,175	829	10.1	7259	598	10.8	55,155
10	11,005	556	32.9	7857	618	−3.4	59,668
11	11,561	603	−8.3	8475	649	−5.0	63,319
12	12,164	304	49.6	9124	428	34.1	67,220
13	12,468	278	8.5	9552	422	1.2	69,480
14	12,746	N/A	N/A	9974	N/A	N/A	71,644

**Table 3:** Femoral length and corpus width of selected *Hyposaurus* femora in mm. NJSM = New Jersey State Museum. YPM = Yale Peabody Museum. FL = Femoral length, distance from the most proximal point on the proximal articular surface of the bone to the most distal point on the lateral distal condyle. CW = Corpus width, the minimum width at the mid-shaft.

ID	Specimen No.	FL	CW
*Hyposaurus rogersii*	NJSM 23368	202.0	20.0
*Hyposaurus rogersii*	NJSM 12293	250.0	27.15
*Hyposaurus rogersii*	NJSM 11069	Missing epiphyses	35.45
*Hyposaurus natator*	YPM 985	250.0	23.0
*Hyposaurus natator*	YPM 753	285.0	28.0

## Data Availability

The data presented in this study are available in Skeletochronology and Paleohistology of *Hyposaurus rogersii* (Crocodyliformes, Dyrosauridae) from the Early Paleogene of New Jersey, USA, and Supplementary Tables.

## Supplementary Materials

The following are available online at https://www.mdpi.com/article/10.3390/ani11113067/s1, Table S1: New Jersey State Museum (NJSM) 23368 Medullary Cavity Measurements by Element to the nearest micron, Table S2: Values obtained from NJSM 23368 Right Humerus slide 1 and Alligator MOR OST-1647 Left Humerus (where noted) used in Skeletochronology models (to the nearest micron), Table S3: Calculation of Mean Percentage Centripetal Increase Factor for the Right Humerus of NJSM 23368, calculated from measurements taken from slide 1 (Table 1), Table S4: Mean Percentage Increase Retrocalculation of CGMs for the right humerus of NJSM 23368, radius a (to the nearest micron), Table S5: Parabolic model retrocalculation of missing CGMs along the main axis of right humerus of NJSM 23368 (to the nearest micron), Table S6: Values obtained from NJSM 23368 Right Femur slide 1 and Alligator MOR OST-1647 Right Femur (where noted) used in Skeletochronology models (to the nearest micron), Table S7: Calculation of Mean Percentage Centripetal Increase Factor for the Right Femur of NJSM 23368, calculated from measurements taken from slide 1 (Table 2), Table S8: Mean Percentage Increase Retrocalculation of CGMs for the right femur of NJSM 23368, radius a+ (to the nearest micron), Table S9: Parabolic model retrocalculation of missing CGMs along the major axis of the right femur of NJSM 23368 (to the nearest micron).
