# Supplementary material for: Skeletochronology and Paleohistology of Hyposaurus rogersii (Crocodyliformes, Dyrosauridae) from the Early Paleogene of New Jersey, USA
Source: Animals (Basel). 2021 Oct 27;11(11):3067. doi: 10.3390/ani11113067 (PMC8614569; doi:10.3390/ani11113067)
Supplement: Supplementary file 1 [file animals-11-03067-s001.zip › animals-1420238-supplementary.pdf]

animals

MDPI

Supplementary Table S1. New Jersey State Museum (NJSM) 23368 Medullary Cavity Measurements by Element to the nearest micron. Diameters measured from clearly identifiable bone to clearly identifiable bone across the cavity (obscure areas considered part of cavity), then divided by two to obtain the radii. The left femur has no strut in its medullary cavity, so the area measurements are the same for the relevant columns. Percentages obtained by multiplying the medullary cavity area by 100 and dividing by the area of the entire cross section (area of cortex plus area of cavity, including strut when present). CPL=Circularly Polarized Light.

Element	Slide	Scan	Radius a	Radius b	Circumference	Area without strut (µm^2)	Area, including strut (µm^2)	Area of cortex (µm^2)	Percentage of the element transverse sectional area occupied by medullary cavity
Right Humerus	1	1	3199	2473	17893	22455610	26349510	286538115	8.5
Left Humerus	3	3a	3174	2628	18265	23319332	28198383	289665515	9.0
Right Femur	1	1 CPL	3266	2473	18117	18818314	26980140	390505013	6.5
Left Femur	2	2 CPL	3478	2262	18234	27846676	27846676	390954452	6.6

Supplementary Table S2. Values obtained from NJSM 23368 Right Humerus slide 1 and Alligator MOR OST-1647 Left Humerus (where noted) used in Skeletochronology models (to the nearest micron). MOR OST=Museum of the Rockies, Osteology Collection.

Description	Value
Major Axis (a-) Broadest Band/Interval	818
Major Axis (a+) Broadest Band/Interval	866
Major Axis (a-) Average Band/Mean interval	453
Major Axis (a+) Average Band/Mean interval	566
Major Axis (a-) Penultimate interval	388
Major Axis (a+) Penultimate interval	368
Major Axis (a-) Mean Percentage Centripetal Increase Factor	20.65
Major Axis (a+) Mean Percentage Centripetal Increase Factor	24.00
Alligator MOR OST-1647 Left Humerus radius a	708
Alligator MOR OST-1647 Left Humerus radius b	558
Distance from neonate MOR OST-1647 Left Humerus periosteal surface to 1st CGM in NJSM 23368 Right Humerus along radius a-	2539
Distance from neonate MOR OST-1647 Left Humerus periosteal surface to 1st CGM in NJSM 23368 Right Humerus along radius a+	3914
Broadest band Retrocalculation of CGMs Lost to Remodeling (Distance from 1st preserved CGM to neonate periosteal surface divided by thickest interval preserved), radius a-	3.10
Broadest band Retrocalculation of CGMs Lost to Remodeling (Distance from 1st preserved CGM to neonate periosteal surface divided by thickest interval preserved), radius a+	4.52
Penultimate interval Retrocalculation of CGMs Lost to Remodeling (Distance from 1st preserved CGM to neonate periosteal surface divided by thickness of penultimate interval), radius a-	6.55
Penultimate interval Retrocalculation of CGMs Lost to Remodeling (Distance from 1st preserved CGM to neonate periosteal surface divided by thickness of penultimate interval), radius a+	10.62
Mean interval Retrocalculation of CGMs Lost to Remodeling (Distance from 1st preserved CGM to neonate periosteal surface divided by mean interval), radius a-	5.61
Mean interval Retrocalculation of CGMs Lost to Remodeling (Distance from 1st preserved CGM to neonate periosteal surface divided by mean interval), radius a+	6.91

Supplementary Table S3. Calculation of Mean Percentage Centripetal Increase Factor for the Right Humerus of NJSM 23368, calculated from measurements taken from slide 1 (Table 1).

Centripetal Percentage Increase	Radius a-	Radius a+
1	86.96	93.24
2	1.66	44.41
3	11.44	7.02
4	18.77	27.60
5	10.62	19.15
6	-50.67	-47.47
7	36.20	77.62
8	20.27	-39.07
9	6.35	33.51
10	64.94	N/A
11	N/A	N/A
Sum	206.53	216.00
Mean Percentage Centripetal Increase Factor	20.65	24.00

Supplementary Table S4. Mean Percentage Increase Retrocalculation of CGMs for the right humerus of NJSM 23368, radius a (to the nearest micron). As can be seen by comparing the last row to the three above it, only 2 CGMs are likely to have been lost—but no more than 3—in radius a-, while only 3 to 4 CGMs are likely to have been lost in a+.

Mean Percentage Increase extrapolation calculation of missing CGMs.	Radius a-	Radius a+
Thickness of first missing CGM interval	987	815
Thickness of second missing CGM interval	1191	1010
Thickness of third missing CGM interval	1436	1252
Thickness of fourth missing CGM interval	N/A	1553
Thickness of fifth missing CGM interval	N/A	1926
Sum of above thicknesses (Only 2 CGMs Retro Calculated)	2177	1825
Sum of above thicknesses (3 CGMs Retro Calculated)	3614	3077
Sum of above thicknesses (4 CGMs Retro Calculated)	N/A	4630
Distance from 1st preserved CGM to neonate periosteal surface	2539	3914

Supplementary Table S5. Parabolic model retrocalculation of missing CGMs along the main axis of right humerus of NJSM 23368 (to the nearest micron). Only 5 to 6 intervals are likely to be missing on radius a- according to this model, while 6 intervals are likely to be missing in radius a+. RetrocCGMs=Retrocalculated Cyclical Growth Marks.

Missing CGM number	Thickness, radius a-	Thickness, radius a+
1	496	492
2	466	808
3	388	455
4	285	866
5	577	726
6	522	569
Sum of RetrocCGMs 1 through 5	2211	3346
Sum of RetrocCGMs 1 through 6	2733	3916
Distance from 1st preserved CGM to neonate periosteal surface	2539	3914

Supplementary Table S6. Values obtained from NJSM 23368 Right Femur slide 1 and Alligator MOR OST-1647 Right Femur (where noted) used in Skeletochronology models (to the nearest micron).

Description	Value
Major Axis (a+) Broadest Band/Interval	1252
Major Axis (a+) Average Band/Mean interval	669
Major Axis (a+) Penultimate interval	304
Major Axis (a+) Mean Percentage Centripetal Increase Factor	21.46
Alligator MOR OST-1647 Right Femur radius a	664
Distance from neonate MOR OST-1647 Right Femur periosteal surface to 1st CGM in NJSM 23368 Right Femur along radius a+	4056
Broadest band Retrocalculation of CGMs Lost to Remodeling (Distance from 1st preserved CGM to neonate periosteal surface divided by thickest interval preserved), radius a+	3.24
Penultimate interval Retrocalculation of CGMs Lost to Remodeling (Distance from 1st preserved CGM to neonate periosteal surface divided by thickness of penultimate interval), radius a+	13.34
Mean interval Retrocalculation of CGMs Lost to Remodeling (Distance from 1st preserved CGM to neonate periosteal surface divided by mean interval), radius a+	6.06

Supplementary Table S7. Calculation of Mean Percentage Centripetal Increase Factor for the Right Femur of NJSM 23368, calculated from measurements taken from slide 1 (Table 2).

Centripetal Percentage Increase	Radius a+
1	9.26
2	98.31
3	-7.69
4	49.07
5	11.18
6	-35.75
7	109.57
8	-37.76
9	62.00
10	-79.01
11	56.86
12	Not Preserved
Sum	236.03
Mean Percentage Centripetal Increase Factor	21.46

Supplementary Table S8. Mean Percentage Increase Retrocalculation of CGMs for the right femur of NJSM 23368, radius a+ (to the nearest micron). According to this model, only 5 CGMs are likely missing, certainly no more than 6.

Description	Value
Thickness of first missing CGM interval	501
Thickness of second missing CGM interval	608
Thickness of third missing CGM interval	738
Thickness of fourth missing CGM interval	897
Thickness of fifth missing CGM interval	1089
Thickness of sixth missing CGM interval	1323
Sum of above thicknesses (Only 2 CGMs Retro Calculated)	1109
Sum of above thicknesses (3 CGMs Retro Calculated)	1847
Sum of above thicknesses (4 CGMs Retro Calculated)	2744
Sum of above thicknesses (5 CGMs Retro Calculated)	3833
Sum of above thicknesses (6 CGMs Retro Calculated)	5157
Distance from 1st preserved CGM to neonate periosteal surface	4056

Supplementary Table S9. Parabolic model retrocalculation of missing CGMs along the major axis of the right femur of NJSM 23368 (to the nearest micron). Only 5 CGMs are missing according to this model.

Description	Value
1st missing interval	263
2nd missing interval	1252
3rd missing interval	773
4th missing interval	1242
5th missing interval	592
6th missing interval	922
Sum of RetrocCGMs 1 through 4	3529
Sum of RetrocCGMs 1 through 5	4122
Sum of RetrocCGMs 1 through 6	5044
Distance from 1st preserved CGM to neonate periosteal surface	4056
